# Increasing Specificity of Correlate Research: Exploring Correlates of Children’s Lunchtime and After-School Physical Activity

**DOI:** 10.1371/journal.pone.0096460

**Published:** 2014-05-08

**Authors:** Rebecca M. Stanley, Kate Ridley, Timothy S. Olds, James Dollman

**Affiliations:** 1 Exercise for Health and Human Performance Group, School of Health Sciences, University of South Australia, Adelaide, South Australia, Australia; 2 Sport, Health and Physical Education Group, School of Education, Flinders University, Adelaide, South Australia, Australia; 3 Health and Use of Time Group, School of Health Sciences, University of South Australia, Adelaide, South Australia, Australia; Purdue University, United States of America

## Abstract

**Background:**

The lunchtime and after-school contexts are critical windows in a school day for children to be physically active. While numerous studies have investigated correlates of children’s habitual physical activity, few have explored correlates of physical activity occurring at lunchtime and after-school from a social-ecological perspective. Exploring correlates that influence physical activity occurring in specific contexts can potentially improve the prediction and understanding of physical activity. Using a context-specific approach, this study investigated correlates of children’s lunchtime and after-school physical activity.

**Methods:**

Cross-sectional data were collected from 423 South Australian children aged 10.0–13.9 years (200 boys; 223 girls) attending 10 different schools. Lunchtime and after-school physical activity was assessed using accelerometers. Correlates were assessed using purposely developed context-specific questionnaires. Correlated Component Regression analysis was conducted to derive correlates of context-specific physical activity and determine the variance explained by prediction equations.

**Results:**

The model of boys’ lunchtime physical activity contained 6 correlates and explained 25% of the variance. For girls, the model explained 17% variance from 9 correlates. Enjoyment of walking during lunchtime was the strongest correlate for both boys and girls. Boys’ and girls’ after-school physical activity models explained 20% variance from 14 correlates and 7% variance from the single item correlate, “I do an organised sport or activity after-school because it gets you fit”, respectively.

**Conclusions:**

Increasing specificity of correlate research has enabled the identification of unique features of, and a more in-depth interpretation of, lunchtime and after-school physical activity behaviour and is a potential strategy for advancing the physical activity correlate research field. The findings of this study could be used to inform and tailor gender-specific public health messages and interventions for promoting lunchtime and after-school physical activity in children.

## Background

Researchers, policy-makers and health professionals are faced with a significant challenge in promoting physical activity (PA) among youth populations within a technology-saturated society. To promote PA, there is a need to better understand the factors that influence children’s choice between active and sedentary pursuits [1], [2].

PA is a complex behaviour that is typically characterised by type (or mode), intensity, frequency and duration [3]. Recently, researchers have been exploring PA from a relatively new and subsequently less frequently studied ‘context’ perspective [3], [4]. The context can be considered as a multi-dimensional acknowledgement of all the characteristics of PA and the circumstances in which PA occurs [3], [4]. Context in essence ‘personalises’ the PA behaviour to a particular person, time, place and activity type. By contextualising PA behaviour, PA correlates become specific and multi-dimensional, rather than generic and one dimensional.

Two time contexts that have been identified as important contributors to children’s daily PA are the lunchtime and after-school periods. Both these time periods are characterized by their discretionary nature (i.e. children can choose to be active or inactive). Lunchtime, also referred to as ‘recess’ in a number of studies, is defined as the primary, regularly scheduled discretionary period where all children have equal opportunity for unstructured activity on a school day, regardless of sex, ethnicity and socio-economic background [5]. Lunchtime play usually takes place outdoors (weather permitting), a location where children are more likely to be physically active [6]–[8]. Accordingly, the lunchtime period provides children with equal opportunities to develop physical competence, health-related fitness, personal and social responsibility, and enjoyment of PA, thereby contributing to the development of healthy life-long PA patterns [5], [9]. For some groups, this may be the only regular opportunity for discretionary PA [10]. Studies have found that the lunchtime period can contribute up to 68% of children’s recommended daily moderate-to-vigorous PA (MVPA) [11]. However, this percentage contribution can be as low as 7% [12]. The factors that contribute to this variance are not well understood.

The after-school time period is unique as it is a time when children have discretion over how they use their time away from the constraints of school and parental curfews [13]. This period (typically defined as 3∶00–6∶00 pm [6], [14], [15]), can account for 21% to 48% of children’s daily MVPA [15], [16] and therefore is critical to children’s overall participation. It has been suggested that the after-school period defines a child’s propensity for PA [14] as those children who report higher incidence of active play during the after-school period, particularly outdoors, are more active overall and active at a higher intensity than those who report a lower incidence of after-school active play [6], [8]. Katzmarzyk et al. [17] suggest that those who choose to be active after school are more likely to have limited time to devote to less active pursuits, such as TV viewing.

There is a range of behavioural theories and models used to identify correlates of PA and to predict, explain and induce change in PA behaviour [3], [18]. While some examine intrapersonal correlates of children’s PA, ecological frameworks take a broader approach, emphasising that PA behaviour is influenced by the direct or indirect interaction of correlates at multiple levels, including person and social, physical, cultural and institutional environments [19], [20].

It has been proposed that in order to maximise predictive capacity of behavioural models to assess PA within specific contexts, the correlate and criterion (PA behaviour) should be measured at the same level of specificity in regards to time, place and activity type (e.g. enjoyment of PA at lunchtime [correlate] and lunchtime PA [behaviour]). In a selective review, Giles-Corti et al. [4] found that the predictive capacity of ecological models appeared to improve when the measured environmental correlates more closely matched the behaviour of interest and the setting in which the behaviour took place. In addition, Humpel et al. [21] found that the predictive capacity of ecological models could be improved if higher specificity was incorporated into the measurement of context-specific behaviour. Emerging research is beginning to address the challenge of defining context-specific physical activity correlates and examining the impact of the context on physical activity behaviour in children [22]–[24]. This study contributes to the evidence by investigating the correlates of children’s objectively measured lunchtime and after-school PA using purposely developed context-specific correlate questionnaires.

## Methods

### Ethics Statement

Ethical approval was obtained from the University of South Australia Human Research Ethics Committee, Department of Education and Children Services (DECS) and the South Australian Commission for Catholic Schools (SACCS). Data will be made available upon request.

### Participants and Sampling

Purposive sampling was used to recruit children in Grades 5, 6 and 7, who were aged between 10 and 14 years. A list of South Australian schools was stratified and divided into bands according to the ‘School Card Register’ (SCR) (0–19, 20–39, 40–59 and 60–100%). The SCR is the percentage of students in a school whose families receive government support to meet the costs of school attendance, and is therefore an inverse indicator of socio-economic status (SES) at the school level. Four schools were randomly sampled from each SCR band resulting in a total of 16 schools invited to participate. Of the 16 invited schools, 10 agreed to participate (62.5%). There were no statistically significant differences found in SES between schools who agreed to participate in the study and those who declined.

Published regression models tend to explain approximately 15% of the total explained variance in PA [22]. To detect 15% of the variance with a power of 0.8 and a significance level of 0.05 for a maximum of 50 potential correlates, a sample size of 237 was required. To allow for incomplete data, an additional 10% was sampled [25], which increased the target sample size to 261. Across the 10 schools, 789 children were invited to take part with 477 providing assent along with written consent from a parent or care giver (60%). A total of 423 participants provided at least one valid dataset (i.e. one questionnaire along with the corresponding time-specific accelerometer data) for inclusion in analyses.

### Measures

#### Youth Physical Activity Survey for Specific Settings (Y-PASS)

Correlates of PA were assessed using the computer-delivered lunchtime and after-school “Youth Physical Activity Survey for Specific Settings” (Y-PASS) questionnaires, two customised context-specific correlate questionnaires that measure potential correlates of children’s lunchtime and after-school PA from a social-ecological perspective [19], [26]. The original pool of items was generated from a comprehensive systematic review of the quantitative correlates literature [27] and focus groups conducted with 54 South Australian children aged 10 to 14 years [28], [29]. The Y-PASS questionnaires were reviewed by a panel of experts with expertise in children’s PA, questionnaire development and correlates of PA and subsequently pilot tested with a sample of South Australian children from Grades 5, 6 and 7 to assess content, usability and design characteristics. An exploratory factor analysis was conducted to identify composite correlate variables and single item correlate variables. The lunchtime questionnaire contained 44 specifically worded items, incorporating nine intrapersonal correlates (e.g. barrier self-efficacy, behavioural attitude/belief), three sociocultural correlates (e.g. peer influence, teacher influence) and six physical environmental/policy correlates (e.g. access to space, access to equipment). An example of a specifically worded lunchtime item was “I like to walk around at lunchtime”. The after-school questionnaire contained 100 items specifically worded for the after-school context and included 23 intrapersonal correlates (e.g. behavioural attitudes/beliefs about organised sports and activities), 12 sociocultural correlates (e.g. parental barriers, license to be active, social support) and 12 physical environmental/policy correlates (e.g. weather, access to equipment, safety). An example of a specifically worded after-school Y-PASS item was “My parents are too busy to play with me after school”. The psychometric properties (factorial structure, internal consistency and test-retest reliability) of the Y-PASS questionnaires have been tested and are presented in Table S1.

#### Physical activity

Lunchtime and after-school PA was objectively measured with the ActiGraph GT3X and GT3X+ accelerometers (ActiGraph, LLC; Fort Walton Beach, FL). The different ActiGraph models have been shown to have acceptable validity and inter-instrument reliability for quantifying PA in children and adolescents [30]–[32]. The agreement between the vertical axis of GT3X and GT3X+ has recently been evaluated in a laboratory study in children and adolescents aged 7–18 years and found to be highly comparable for vertical axis counts (ICC = 0.994) and estimated time spent in MVPA (ICC = 0.996) [32]. Actigraphs were worn on the right hip using an elastic belt and set to collect uniaxial data in epochs of 15 seconds.

#### Anthropometric measures

Height and weight were measured using protocols of the International Society for the Advancement of Kinanthropometry (ISAK) [33]. BMI z-scores were calculated using the U.S. Centre for Disease Control and Prevention reference standards [34].

#### Demographic measures

Each child provided details of demographic characteristics, including sex, date of birth and postcode of residence. Residential postcodes were used to determine the SEIFA (Socio-Economic Index for Areas) score, an index developed by the Australian Bureau of Statistics (ABS) to identify SES levels [35].

### Procedure

Data collection occurred between May and July 2011. Children wore the accelerometers for five school days (i.e. Monday to Friday) and completed the two Y-PASS questionnaires in a school computer room during the school week. Questionnaire administration was standardised and the completion order randomised.

### Data Treatment

#### Y-PASS

Each questionnaire item response was assigned a number: Disagree a lot = 1, Disagree a little = 2, Neither disagree nor agree = 3, Agree a little = 4, Agree a lot = 5. Some items are negatively related to PA and were reverse coded. Factor scores were derived by averaging response scores for items representing each factor. For individual correlate items, the score was the response score provided by the participant.

Despite attempts to minimise item non-response through the use of an online format, there were a very small number of responses missing. The lunchtime questionnaire had one missing response from one participant, the after-school questionnaire had four missing responses from three participants, equating to less than 1% of all responses. The missing responses were at random with no general pattern across the responses. Reasons for missing data were unknown but it may have been due to software glitches in the Survey Gizmo system (Widgix, 2005–2010). ‘Hot deck’ imputation was conducted on items with incomplete responses – a commonly used procedure which assigns a value for a missing item based on the responses from comparable respondents in the sample (i.e. respondents reflecting similar demographic and response characteristics) [36], [37]. The hot deck method has been found to be the most accurate data imputation technique, according to one study comparing six different data imputation techniques [38].

#### Accelerometry

Accelerometer data were downloaded with ActiLife Software Version 5.6 (ActiGraph). Using customised software (developed by Deakin University, Melbourne, Australia) accelerometer data were screened for non-wear time defined as 20 minutes of consecutive zero and setting the acceptable upper limit of 15,000 counts per minute [39]. For lunchtime and after-school periods to be considered valid, children were required to provide counts for at least 50% of a lunchtime period and after-school period [40]and to provide at least two days of valid lunchtime data and three days of valid after-school data. These criteria were identified using pilot accelerometer data and calculating intraclass correlations (ICC) to determine how many days were required to reliably capture ‘typical’ lunchtime and after-school MVPA at a precision of 80%. Two days of lunchtime MVPA data yielded ICCs of 0.90 for both boys and girls, while three days of after-school data yielded ICCs of 0.86 and 0.89 for boys and girls, respectively. Accelerometer data were summarised as time spent in MVPA, expressed as a percentage of the monitored wear time during the lunchtime or after-school periods, averaged over valid days. MVPA was derived from age-specific cut-points and using a 4 MET definition [41].

### Statistical Analysis

The Statistical Package for the Social Sciences (SPSS) Version 19.0 (SPSS, Chicago, IL) and XLSTAT (Addinsoft SARL, Germany) were used to conduct analyses. As schools had different lunchtime durations and end-of-school times, the percentage of lunchtime and after-school MVPA was used as the dependent variable. The independent variables were the correlate factors and items, age, BMI z-score and SEIFA score (total independent variables = 21 [lunchtime] and 49 [after-school]). Clustering at the school level was checked prior to analyses by testing the intraclass correlations (ICC) in lunchtime and after-school physical activity among schools. No significant difference in physical activity levels was found between schools for lunchtime physical activity (ICC = −0.12, p = 0.78) or afterschool physical activity (ICC = −0.03, p = 0.76) and therefore, controlling for clustering in schools in the analyses was not required [42]. Multicollinearity between independent variables was found in the after-school Y-PASS questionnaire, which violates the assumptions for Multiple Regression analysis [42]. Also, the sample size for the after-school gender-specific analyses was found to be lower than required. Therefore, Correlated Components Regression for linear regression models (CCR-LM) was chosen as a more appropriate statistical analysis test for identifying the correlates for lunchtime and after-school PA [43] as it accounts for potential interrelationships among the correlate variables [44] and insufficient sample sizes [43]. An alpha level of 0.05 was used to infer statistical significance and analyses were conducted for boys and girls separately.

## Results

### Participant Characteristics

Table 1 summarises the demographic characteristics and lunchtime and after-school PA levels of the sample.

**Table 1:** Characteristics of the sample.

	Boy (n = 200)		Girl (n = 223)		Total (n = 423)	
Age [mean (SD)]	11.72 (0.78)		11.74 (0.81)		11.73 (0.80)	
Age Range	10.1–13.3		10.2–13.5		10.1–13.5	
**Grade level**	**n**	**%**	**n**	**%**	**n**	**%**
Grade 5	51	26.0	59	27.0	110	26.0
Grade 6	84	42.0	88	40.0	172	41.0
Grade 7	65	33.0	76	34.0	141	33.0
**Socio-economic status** a	**n**	**%**	**n**	**%**	**n**	**%**
Low SES (SEIFA ≤973)	103	52.0	118	53.0	221	52.0
High SES (SEIFA >973)	97	48.0	105	47.0	202	48.0
SEIFA [mean (SD)]	971.6 (75.9)		965.4 (79.7)		968.3 (77.9)	
SEIFA Range	788–1121		788–1136		788–1136	
**BMI Classification**	**n**	**%**	**n**	**%**	**n**	**%**
Thin	12	6.0	10	4.5	22	5.2
Normal	131	65.5	155	69.5	286	67.6
Overweight	39	19.5	41	18.4	80	18.9
Obese	10	5.0	11	4.9	21	5.0
Missing data	8	4.0	6	2.7	14	3.3
**PA**	**n**	**%**	**n**	**%**	**n**	**%**
Proportion of lunchtime spent in MVPA (%)	200	34.7 (14.0)	223	21.3 (9.8)	423	27.6 (13.7)
Contribution of lunchtime MVPA to recommended daily PA (%)	200	20.9 (9.2)	223	12.6 (6.1)	423	16.5 (8.8)
Proportion of the after-school period spent in MVPA (%)	186	13.5 (7.2)	216	10.6 (5.6)	402	11.9 (6.5)
Contribution of after-school MVPA to recommended daily PA (%)	186	36.9 (20.1)	216	29.2 (15.7)	402	32.8 (18.3)

a The nation-wide average SEIFA score is 1000, with a standard deviation of 100 [35].

### Correlates of Lunchtime and After-school PA

The correlates, unstandardised and standardised regression coefficients, R^2^ values and the cross-validation R^2^ values for the total models, along with tolerance intervals, are presented for boys’ and girls’ PA in Tables 2 and 3.

**Table 2:** Results of the CCR analyses to explain lunchtime PA in boys (n = 186) and girls (n = 215) using the lunchtime Y-PASS questionnaire.

Correlate	Unstandardised coefficients	Standardised coefficients	R-squared	Cross-validation adjusted R-squared (95% Confidence Interval [Coefficient of Variation])
**Boys**				
Age	−3.57	−0.20	0.25	0.16 (±0.02)
SEIFA	0.03	0.19		
Behavioural attitude/belief	2.84	0.14		
I like to walk around at lunchtime.	−3.69	−0.32		
Peer influence	3.61	0.19		
Our school play area has painted lines onthe ground (e.g. hopscotch and4-square) to help me be active at lunchtime.	−2.58	−0.23		
**Girls**				
Age	−1.13	−0.09	0.17	0.12 (±0.009)
Barrier self-efficacy	1.14	0.07		
Perceived self-efficacy	1.63	0.10		
I can still be active at lunchtime even ifI am wearing my school uniform.	0.57	0.07		
I like to walk around at lunchtime.	−0.97	−0.11		
I really like doing PE at school.	0.93	0.08		
I always have the energy to be active at lunchtime.	0.62	0.06		
Peer influence	1.27	0.10		
Access to facilities/equipment	0.78	0.07		

**Table 3:** Results of the CCR analyses to explain after-school PA in boys (n = 179) and girls (n = 208) using the after-school Y-PASS questionnaire.

Correlate	Unstandardised coefficients	Standardised coefficients	R-squared	Cross-validation adjusted R-squared (95% Confidence Interval [Coefficient of Variation])
**Boys**				
Behavioural attitudes/beliefs (organised sports/activities)	0.53	0.06	0.20	0.15 (±0.01)
Behavioural attitudes/beliefs (non-organised activities)	0.51	0.06		
Barriers self-efficacy	0.47	0.05		
Support seeking/social norm	0.33	0.05		
Perceived competence	0.31	0.04		
Perceived barriers	0.39	0.04		
I do an organised sport or activity after schoolbecause I want to improve my skills.	0.30	0.04		
I do an organised sport or activity after schoolbecause I want to meet new people.	0.27	0.04		
I do an organised sport or activity after schoolbecause it gets you fit.	0.30	0.05		
Social support	0.50	0.06		
I am not active after school because I haveno one to play with.	0.34	0.06		
Weather	0.39	0.05		
Access to facilities/equipment	0.43	0.04		
I have enough time to do an organised sportor activity after school.	0.30	0.04		
**Girls**				
I do an organised sport or activity after school becauseit gets you fit.	1.55	0.26	0.07	0.04 (±0.02)

From 21 potential lunchtime Y-PASS correlates entered into the regression model, six correlates of lunchtime PA were identified for boys, explaining 25% of the variance, of which four correlates related to the intrapersonal domain, one related to the sociocultural domain and one related to the physical environment/policy domain of the social-ecological framework. For girls, nine correlates were identified, explaining 17% of the variance (Table 2). Similar to the boys, majority of the correlates were intrapersonal (seven correlates), with one sociocultural and one physical environment/policy correlate identified in the models. The single item correlate, “enjoyment of walking around at lunchtime”, was the strongest negative correlate (β = −0.32) for boys, followed by “Painted lines on the ground in the school play area” (β = −0.23). The positive correlates of boys’ lunchtime MVPA were “Peer influence” (β = 0.19), “SEIFA” (β = 0.19) and “Behavioural attitudes and beliefs” (β = 0.14). For girls, lunchtime physical activity was also strongly negatively associated with “Enjoyment of walking around at lunchtime” (β = −0.11) and = positively associated with “Peer influence” (β = 0.10). The single item correlates, “I can still be active at lunchtime even if I am wearing my school uniform” (β = 0.07) and “Always having energy to be active at lunchtime” (β = 0.06), were also identified as significant correlates for girls but not for boys.

Results for after-school PA are presented in Table 3. From the 49 potential after-school Y-PASS correlates entered into the regression model, 14 correlates were identified for boys, accounting for 20% of the variance, with nine correlates relating to the intrapersonal domain, two correlates relating to the sociocultural domain and three relating the physical environment/policy domain of the social-ecological framework. All the correlates were positively associated with after-school PA and were of relatively similar importance (β = 0.04–0.06). Of the correlates identified in the model, the most important for boys’ after-school physical activity was “Social support” and the least important correlates were “Perceived competence” and “Access to facilities and equipment”. For girls, “I do an organised sport or activity after school because it gets you fit” was the only significant intrapersonal correlate, explaining 7% of the variance in after-school MVPA.

## Discussion

Obtaining a better understanding of the correlates of children’s PA in specific contexts is crucial for advancing the PA and correlate research fields [4], [27]. This study provides important insights into the correlates of children’s objectively measured lunchtime and after-school PA. Notably, boys’ and girls’ PA were influenced differently during the same context and across contexts.

The strongest correlate for both boys’ and girls’ lunchtime PA was the single item correlate “I like to walk around at lunchtime” and this was a negative association. The negative association may be attributable to lower PA levels of children who agreed with this statement. In this context, walking around the school yard may have not reached the MVPA threshold according to the Freedson cutpoint [41]. Children who enjoyed walking around at lunchtime spent less time in MVPA during lunchtime (i.e. 24% of lunchtime in MVPA) compared to children who disagreed with this statement (i.e. 37% of lunchtime in MVPA). Walking around at lunchtime may be an opportunity for children to engage in other activities, such as eating or socialising with friends, which are more difficult to perform during more vigorous activity. This walking behaviour should not necessarily be discouraged because it is an alternative activity to sedentary activities such as sitting, which has been identified as an independent risk factor for chronic disease [45], [46]. However, as much of the evidence associating PA and health benefits in children is at the moderate to vigorous intensity, it is important that children have a balance between lower intensity, health promoting activities and more vigorous activities across different contexts, while aiming to meet recommended PA guidelines [47].

Enjoyment of PE at school was also identified as a strong intrapersonal correlate for girls only. While this correlate may seem unusual for lunchtime PA, previous research has found that PE enjoyment is associated with PA behaviour in other contexts [22], [48], [49]. These studies posit that children who enjoy activities and are self-determined will transfer behaviours from one context to others [48], [49]. It may be that children who enjoy PE lessons are more likely to have better motor skills, which may facilitate engagement in other activities, particularly more organised activities [50].

In this study, “I can still be active at lunchtime even if I am wearing my school uniform” was positively associated with girls’ lunchtime MVPA. Girls have previously reported that having to wear school dresses and heavy school shoes deterred them from physically active games, such as kicking a football or playing basketball [28]. Further exploration into the influence of different types of school uniforms worn by the children (e.g. dresses versus shorts/trousers and t-shirts, heavy school shoes versus sneakers) on PA would yield a more in-depth understanding of this issue and could potentially lead to important changes in school uniform policy.

The item “I do an organised sport or activity after school because it gets you fit” was purposely developed for the Y-PASS questionnaire because it was perceived to be one of the most important factors that influenced after-school PA by both boys and girls in focus group discussions [28]. Interestingly, this was the only correlate identified in the whole model for girls while other beliefs about organised sports and activities, including improving skills and meeting new people, also correlated with after-school MVPA for boys. Studies have found that all these factors are strong motivators for engaging in organised sports [51], and may provide avenues to promote and increase PA levels.

In both contexts, sociocultural influences were identified for boys’ and girls’ lunchtime PA and boys’ after-school PA. The most important sociocultural correlate for lunchtime PA was “peer influence”. Similarly, social influences including peer and parent support were correlates of boys’ after-school activity. Ecological models postulate that influences most proximal to the target group will have the strongest effect on the desired behaviour [19], [26], demonstrated by the identification of peer rather than teacher influence as a correlate of lunchtime PA, and parental and peer support as correlates of after-school PA in the current study. To date, no other lunchtime-specific studies have assessed the relationship of peer influence with children’s lunchtime PA [27]. In two after-school studies, Hohepa et al. [52] and Ommundsen et al. [22] also found that parental and peer support were key correlates of youth after-school PA. While social support appears to be important for both children’s lunchtime and after-school PA, the findings of this study reinforce that the source of effective social support is context-specific.

The perception of whether painted lines on the ground helped children be active at lunchtime was negatively associated with boys’ lunchtime MVPA. This contrasts with previous observational and experimental studies reporting positive associations [53], [54] or no associations [55] with lunchtime PA behaviour. While children may perceive this environmental feature as helping them be active, actual PA may be limited by the time spent waiting for turns due to the popularity of activities (e.g. handball and hopscotch). Based on evidence from observational studies [56], Wechsler et al. [9] concluded that children spend approximately half of their lunch break engaging in physical activities, while the remaining time is spent waiting for a turn or observing others.

In the context of existing literature, the amount of variance explained in the current study appears to be quite low in comparison to other lunchtime and after-school PA studies [22], [57] and this may be partly attributable to shared method variance. For example, Ommundsen et al. [22] used self-report to assess both physical environmental correlates of PA, explaining 55% of lunchtime PA, and 44% of after-school PA. Using self-report methods to measure both correlates and PA may result in systematic over- or under-reporting, leading to inflated correlations and larger total explained variance [58], [59]. In the current study, PA was objectively measured while context-specific correlates were self-reported. Dishman et al. [60] and Lubans et al. [58] also found that correlations between objectively measured PA and self-reported correlates were much lower than previous studies using self-report measures of PA.

The lower variance of girls’ after-school PA explained in the current study is consistent with the correlates literature, whereby girls’ PA is largely unexplained by current theories [58], [61], [62]. Despite developing a targeted social cognitive model for girls’ PA, Lubans et al. [58] were able to explain only 5% of the variance in girls’ PA, with only self-efficacy, school environment and physical self-worth predicting accelerometer counts. Similarly, behavioural models used in a study by Trost et al. [61] only accounted for 10% and 5% of White and African-American girls’ PA respectively.

### Strengths and Limitations

Strengths of this study include a large and diverse sample, objectively measured PA and use of a multi-dimensional theoretical framework to explore the influences of PA behaviour. In addition, sex-specific analyses showed that sex differences exist, highlighting the need for appropriately sensitive correlate measures [63].

A number of limitations to this study need to be acknowledged. Firstly, the cross-sectional nature of this study precludes the evaluation of any causal relationships. Although random sampling was conducted to obtain a representative sample of schools in different SES areas, it is possible that the number of schools selected was not necessarily proportional to the distribution of schools within each SCR band. In addition, classes within each school were not randomly selected and this limits the generalisability of the findings. The predictive capacity of questionnaires is reliant on variation in intrapersonal, sociocultural and physical environmental/policy correlates, as well as PA behaviour [64]. In the current study, low variability in some correlate variables may have limited the explained variance in PA in the models. For example, the school playground tends to be quite structured and relatively homogeneous across schools, with little variation in aspects such as availability of equipment and play spaces (including bitumen areas and grassed fields) and the presence of teacher supervision [65]. This does not imply that the factor is unimportant, rather that it does not explain variance in PA behaviour in the particular sample. In addition, a number of the correlates identified from the Y-PASS questionnaires encapsulate some of the PA outcome variables, e.g. “I do an organised sport or activity after school because it gets you fit”. This may mean that only children engaging in the specific type of PA in question will have an opinion and be able to respond appropriately to the item. Children may disagree with these types of statements because they do not participate in the specific type of activity, or they may participate but disagree with the motivation for participation. It is important to keep this in mind when interpreting the strength of relationship of these types of items with context-specific PA. Finally, while accelerometers are advantageous in reducing reporting bias, technological limitations, such as the inability to detect upper body movement, cycling, and water-based activities are acknowledged. Accelerometer data treatment decisions about cut-points for MVPA can also affect the strength of association between correlates and PA behaviour [66], [67].

### Practical Implications and Future Recommendations

It appears that the predictive utility of the after-school Y-PASS questionnaire is not as effective as the lunchtime Y-PASS, suggesting that the after-school context is more complex with a greater diversity of activity options and environmental settings. These findings demonstrate that there is still further research to be undertaken to improve the prediction of children’s context-specific physical activity and theories used to understand and promote PA. This study provides a platform for the development of future studies.

Future studies could include objective measures of the environment using appropriate methods, such as direct observation or school audits. The after-school period could be broken down into exploring the correlates of different types of behaviours occurring during this period separately, such as after-school active transport, after-school organised sports and activities and after-school non-organised activities, rather than trying to explore correlates of all these types of behaviours in the one questionnaire. This will allow for a much more in-depth exploration of the different types of behaviours occurring in the after-school time period.

There is some reluctance in making major intervention recommendations based on the current findings due to a large percentage of unexplained PA variance [68] and the cross-sectional nature of the data. However, the identified barriers and facilitators of lunchtime and after-school PA in this study could be further researched to better inform the development of appropriately targeted lunchtime and after-school PA interventions. Examples of potential strategies include changing uniform policies or design to be practical and allow ease of movement; modifying PE lessons to enhance enjoyment in girls, employing strategies such as increasing children’s sense of activity choice to better reflect gender equality [69], [70]; strengthening peer relationships in relation to PA; and targeting engagement in after-school organised sports and activities.

As this study demonstrates that boys and girls are influenced differently in different contexts, future research should focus on developing gender-specific theories for understanding context-specific PA behaviour, particularly for girls [58], [61]. Also, research has shown differences in the relationships between correlates and activity level as a function of the type of physical activity assessment technique used [71], [72]. Therefore, theories should be revisited and tested using both self-report and objective measures of PA in different contexts, settings and among different cultures as this may provide new insights into factors influencing context-specific PA behaviour and has important implications for theoretical-based interventions.

## Conclusions

The lunchtime and after-school time periods are two critical windows in a school day for children to accumulate recommended PA levels. It is important to have a clear, meaningful picture of what is influencing children’s PA during these periods so that interventions can be developed that target the most important correlates and the distinct needs of boys and girls. Using a context-specific approach has enabled us to identify the unique features of PA behaviour in specific contexts for boys and girls and underscores the potential value of using specific conceptual frameworks in future correlate research for understanding and examining children’s PA. However, there remains much variance unexplained by the Y-PASS questionnaires, which warrants attention in future research.

## Supporting Information

Table S1 **Psychometric properties of the Y-PASS questionnaires.**
(DOCX)
