# Supplementary material for: Increasing Specificity of Correlate Research: Exploring Correlates of Children’s Lunchtime and After-School Physical Activity
Source: PLoS One. 2014 May 8;9(5):e96460. doi: 10.1371/journal.pone.0096460 (PMC4014506; doi:10.1371/journal.pone.0096460)
Supplement: Table S1 — Psychometric properties of the Y-PASS questionnaires. (DOCX) [file pone.0096460.s001.docx]

### Table S1 – Psychometric properties of the Y-PASS questionnaires

| **Questionnaire** | **Factor** | **Test-retest (ICC)** | **Cronbach alpha** |
| --- | --- | --- | --- |
| **Lunchtime Y-PASS** |  |  |  |
| *Intrapersonal subscale* | Barrier self-efficacy (7 items) | 0.84 (p<0.000) | 0.80 |
|  | Perceived self-efficacy (6 items) | 0.73 (p<0.000) | 0.78 |
|  | Behavioural attitude/belief (5 items) | 0.73 (p<0.000) | 0.78 |
|  | I can still be active at lunchtime even if I am wearing my school uniform. | 0.67 (p<0.000) | - |
|  | I like to walk around at lunchtime. | 0.39 (p=0.06) | - |
|  | I really like doing PE at school. | 0.66 (p<0.000) | - |
|  | I always have the energy to be active at lunchtime. | 0.33 (p=0.11) | - |
|  | I am just as coordinated as kids of my age and gender. | 0.47 (p=0.02) | - |
|  | Making up your own game rules makes playing games at lunchtime more fun. | 0.47 (p=0.02) | - |
| *Sociocultural subscale* | Peer influence (3 items) | 0.70 (p<0.000) | 0.64 |
|  | Teacher influence (2 items) | 0.63 (p=0.001) | 0.64 |
|  | Social barriers (3 items) | 0.57 (p=0.004) | 0.32 |
| *Physical environmental/policy subscale* | Access to facilities/equipment (3 items) | 0.71 (p<0.000) | 0.61 |
|  | Physical environmental/policy barriers (3 items) | 0.66 (p<0.000) | 0.55 |
|  | Access to space (3 items) | 0.60 (p=0.002) | 0.50 |
|  | We have school rules about where we are allowed to be active at lunchtime. | 0.47 (p=0.02) | - |
|  | There are facilities at school, such as playgrounds or ovals, where I can be active at lunchtime. | 0.46 (p=0.02) | - |
|  | Our school play area has painted lines on the ground (e.g. hopscotch and 4-square) to help me be active at lunchtime. | 0.75 (p<0.000) | - |
| **After-school Y-PASS** |  |  |  |
| *Intrapersonal subscale* | Behavioural attitudes/beliefs (organised sports/activities) (7 items) | 0.85 (p<0.000) | 0.77 |
|  | Behavioural attitudes/beliefs (non-organised activities) (6 items) | 0.93 (p<0.000) | 0.82 |
|  | Barriers self-efficacy (6 items) | 0.73 (p<0.000) | 0.80 |
|  | Support seeking/social norm (3 items) | 0.58 (p=0.005) | 0.70 |
|  | Perceived competence (2 items) | 0.73 (p<0.000) | 0.82 |
|  | Perceived barriers (3 items) | 0.62 (p=0.002) | 0.43 |
|  | I wish I could do more organised sports or activities after school than I get a chance to. | 0.72 (p<0.000) | - |
|  | Being active after school is boring. | 0.63 (p=0.002) | - |
|  | I do an organised sport or activity after school because I want to improve my skills. | 0.68 (p=0.001) | - |
|  | I do an organised sport or activity after school because I want to meet new people. | 0.55 (p=0.007) | - |
|  | I ride, walk, skate or scooter to and from places after school because it gets you fit. | 0.37 (p=0.09) | - |
|  | Playing outside after school keeps you healthy. | 0.69 (p<0.000) | - |
|  | I do an organised sport or activity after school because I have nothing else to do. | 0.13 (p=0.58) | - |
|  | I don’t participate in some activities after school because it is only a boys’/girls’ activity. | 0.26 (p=0.24) | - |
|  | I play actively at home or in the neighbourhood after school because it gives me something to do. | 0.57 (p=0.006) | - |
|  | I do an organised sport or activity after school because it gets you fit. | 0.45 (p=0.04) | - |
|  | I am active after school because I don’t want to put on weight. | 0.65 (p=0.001) | - |
|  | I am confident that I can be active after school on most days even if I could watch TV or play video games instead. | 0.48 (p=0.02) | - |
|  | I am confident that I can be active after school on most days even if it is hot or cold outside. | 0.66 (p=0.001) | - |
|  | I am confident that I can be active after school on most days even if I am being bullied. | 0.72 (p<0.000) | - |
|  | I am just as coordinated as kids of my age and gender. | 0.80 (p<0.000) | - |
|  | I am shy about how my body looks. | 0.69 (p<0.000) | - |
|  | I really like doing PE at school. | 0.57 (p=0.005) | - |
| *Sociocultural subscale* | Social support (7 items) | 0.91 (p<0.000) | 0.78 |
|  | Parental barriers (4 items) | 0.55 (p=0.007) | 0.63 |
|  | License to be active (4 items) | 0.85 (p<0.000) | 0.75 |
|  | Parental rules (2 items) | 0.77 (p<0.000) | 0.56 |
|  | Bullying stops me from being active after school. | 0.34 (p=0.13) | - |
|  | I am not active after school because I have no one to play with. | 0.75 (p<0.000) | - |
|  | I have brothers/sisters who play actively with me after school. | 0.41 (p=0.06) | - |
|  | My friends encourage me to be active after school. | 0.33 (p=0.13) | - |
|  | My friends tell me I am doing well at sport. | 0.60 (p=0.003) | - |
|  | My parents are too busy to play with me after school. | 0.58 (p=0.004) | - |
|  | My parents make me help around the house, which stops me from being active after school. | 0.31 (p=0.17) | - |
|  | We have a rule at home that we have to do an organised sport or activity after school. | 0.40 (p=0.07) | - |
| *Physical environmental/policy subscale* | Weather (6 items) | 0.69 (p<0.000) | 0.75 |
|  | Access to facilities/equipment (7 items) | 0.80 (p<0.000) | 0.69 |
|  | Safety (4 items) | 0.75 (p<0.000) | 0.63 |
|  | Access to space (3 items) | 0.75 (p<0.000) | 0.60 |
|  | Time commitments (2 items) | 0.66 (p=0.001) | 0.61 |
|  | Financial barriers (3 items) | 0.60 (p=0.003) | 0.64 |
|  | School bag (2 items) | 0.53 (p=0.01) | 0.54 |
|  | Dog poo on the lawn at home keeps me from being active on the grass after school. | 0.38 (p=0.09) | - |
|  | I have enough time to do an organised sport or activity after school. | 0.80 (p<0.000) | - |
|  | It is safe to play actively near where I live after school. | 0.70 (p<0.000) | - |
|  | There are not enough traffic lights and crossings in my neighbourhood after school. | 0.47 (p=0.03) | - |
|  | There is enough equipment to play actively at home after school. | 0.27 (p=0.23) | - |
